# Effect of DNA Groove Binder Distamycin A upon Chromatin Structure

**DOI:** 10.1371/journal.pone.0026486

**Published:** 2011-10-26

**Authors:** Parijat Majumder, Dipak Dasgupta

**Affiliations:** Biophysics Division, Saha Institute of Nuclear Physics, Kolkata, West Bengal, India; Shantou University Medical College, China

## Abstract

**Background:**

Distamycin A is a prototype minor groove binder, which binds to B-form DNA, preferentially at A/T rich sites. Extensive work in the past few decades has characterized the binding at the level of double stranded DNA. However, effect of the same on physiological DNA, i.e. DNA complexed in chromatin, has not been well studied. Here we elucidate from a structural perspective, the interaction of distamycin with soluble chromatin, isolated from Sprague-Dawley rat.

**Methodology/Principal Findings:**

Chromatin is a hierarchical assemblage of DNA and protein. Therefore, in order to characterize the interaction of the same with distamycin, we have classified the system into various levels, according to the requirements of the method adopted, and the information to be obtained. Isothermal titration calorimetry has been employed to characterize the binding at the levels of chromatin, chromatosome and chromosomal DNA. Thermodynamic parameters obtained thereof, identify enthalpy as the driving force for the association, with comparable binding affinity and free energy for chromatin and chromosomal DNA. Reaction enthalpies at different temperatures were utilized to evaluate the change in specific heat capacity (ΔCp), which, in turn, indicated a possible binding associated structural change. Ligand induced structural alterations have been monitored by two complementary methods - dynamic light scattering, and transmission electron microscopy. They indicate compaction of chromatin. Using transmission electron microscopy, we have visualized the effect of distamycin upon chromatin architecture at di- and trinucleosome levels. Our results elucidate the simultaneous involvement of linker bending and internucleosomal angle contraction in compaction process induced by distamycin.

**Conclusions/Significance:**

We summarize here, for the first time, the thermodynamic parameters for the interaction of distamycin with soluble chromatin, and elucidate its effect on chromatin architecture. The study provides insight into a ligand induced compaction phenomenon, and suggests new mechanisms of chromatin architectural alteration.

## Introduction

Distamycin A (DST) is an oligopeptide antibiotic, biosynthesized by *Streptomyces distallicus*. It is known to bind isohelically to the minor groove of B-DNA at A/T rich sites [1], [2], [3], [4]. The binding takes place due to favorable van der Waals interactions between C-H's of the aromatic pyrroles of DST and adenine C2-H's in the B-DNA minor groove, along with hydrogen bond formation between NH groups of the pyrrole carboxamide rings and N3 of adenine or O2 of thymine [5]. Ligand-DNA complex is further stabilized by electrostatic interactions between the negatively charged phosphate backbone of DNA and the positively charged terminus of the ligand. Binding of DST A to DNA, widens the minor groove by unbending the helix axis and lengthening it by nearly 12–15% [5], [6].

For over decades, DST has been studied as a prototype minor groove binder to understand the structural aspects of ligand- double helical nucleic acid interactions [1], [2], [7]–[11]. Its preference for A/T rich sites has made it a simple, yet effective probe to characterize the behavior of different DNA backbone structures towards DNA binding ligands [12], [13]. However, it is now well accepted that ligand – DNA interactions in the cell have higher level of complexity due to the presence of proteins that are intimately associated with the template DNA. These proteins scaffold the DNA to form a hierarchically packaged assemblage called chromatin. The proteins give it structure and at the same time, regulate its accessibility towards various ligands.

At the cellular level, extensive studies on this molecule have revealed that it inhibits the pathogenesis of vaccinia virus in culture [14]. It specifically enhances the rate of functional complex formation at the promoter, thereby activating transcription initiation [15]. There are also reports suggesting that it inhibits homeodomain-DNA complexes [16], TBP binding and basal *in vitro* transcription [17]. It displaces the essential transcription factors like SRF and MEF2 [18], and inhibits binding of the high mobility group protein HMGA1 to P-Selectin promoter [19]. It also specifically inhibits binding of DNA to nuclear scaffold and histone H1 [20].

Although substantial work has progressed in evaluating the drug potential of the molecule, yet a biophysical characterization of the effect of the molecule on chromatin is still wanting. Till date, reports that elucidate the interaction of DST with chromatin, mainly concern the mode of binding of the drug, emphasizing on its A/T selectivity. The studies include DNAaseI and hydroxyl radical footprinting of DST with nucleosome core particles, reconstituted on *tyr*T DNA fragment, or a cloned synthetic sequence containing phased repeats of (A/T)_4_ [21], [22]. The results show that DST alters the rotational orientation of core DNA, placing the antibiotic on the inward facing surface of the core DNA supercoil.

The present study has two components. First, we have employed isothermal titration calorimetry to evaluate the binding parameters and thermodynamic features (such as change in heat capacity) for the association of DST with soluble chromatin and its components, namely chromatosomes and chromosomal DNA. Chromatosomes are asymmetric mononucleosomal particles containing a single linker histone [23]. Binding studies with chromatosomes and histone-free DNA templates helped us to estimate the binding preference of the molecule. Secondly, the binding studies have been supplemented with Dynamic Light Scattering and Transmission Electron Microscopy of chromatin, dinucleosomes and trinucleosomes. Results from the two types of studies elucidate the effect of the classical minor groove binder DST upon chromatin architecture We have previously employed calorimetry to understand the structural consequences of the interaction of chromatin with an intercalator, sanguinarine, at various chromatin structural levels [24].

## Materials and Methods

### Preparation of distamycin A solution

Distamycin A (Sigma) was dissolved in 5 mM Tris HCl (pH 7.4) containing 20 mM NaCl and the concentration was determined using molar extinction coefficient of 34000 M^−1^ cm^−1^ at 303 nm [25].

### Preparation of chromatin samples and DNA

Soluble chromatin was isolated from the liver of male albino Sprague-Dawley rats, obtained from the Indian Institute of Chemical Biology, Kolkata, India. Rat liver nuclei were isolated as described by Blobel and Potter [26]. Chromatin was prepared from rat liver nuclei by partial digestion with micrococcal nuclease (Sigma) [27], [28]. For preparation of chromatosomes, the micrococcal nuclease digestion time was increased from 30 seconds to 5 minutes. The soluble fraction thus obtained, was purified by centrifugation through a 5–20% linear sucrose density gradient. Di- and trinucleosomes were obtained by size fractionation of rat liver soluble chromatin by sedimentation through 20–30% linear sucrose gradients prepared in 5 mM tris HCl (pH 7.4), 15 mM NaCl, 2 mM EDTA [29]. It is to be noted that chromatosome, di- and trinucleosome samples prepared by such a method, all contain linker histones. Chromosomal DNA was isolated from soluble chromatin by phenol – chloroform – isoamyl alcohol extraction followed by precipitation with isopropanol. Unless otherwise stated, all samples, prior to experiment were dialyzed extensively against 5 mM Tris HCl (pH 7.4) containing 15 mM NaCl and mononucleotide concentrations of the samples were determined spectrophotometrically using the molar extinction coefficient (ε_260_) of 6600 M^−1^ cm^−1^. At the ionic strength used for our experiments, chromatin samples are known to exist as stable extended structures [30].

### Isothermal Titration Calorimetry

Soluble chromatin, chromatosome and chromosomal DNA were individually titrated against DST solution in 5 mM Tris HCl (pH 7.4), 15 mM NaCl. Typically, 1.4 ml of macromolecule (120 µM DNA base), loaded in the calorimetric cell was titrated against 330 µM of the antibiotic solution (20 injections of 9 µl each or 35 injections of 6 µl each, with an initial injection of 1 µl followed by 3 µl) using a 289 µl syringe, rotating at 286 r.p.m. ITC measurements of DST dilution in buffer served as control. Calorimetric titrations were performed at multiple temperatures (10°C, 15°C, 20°C and 25°C for chromatin and chromosomal DNA and 13°C, 17°C, 21°C and 25°C for chromatosome) in a MicroCal VP-ITC microcalorimeter. The resulting thermograms were analyzed using single set of binding sites model of Levenberg – Marquardt non-linear least squares curve fitting algorithm, inbuilt in the MicroCal LLC software. The apparent association constant *K_a_*, site size *n*, and molar heat of binding Δ*H_b_* were obtained using the following relation:
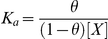
where *θ* = fraction of sites occupied by ligand *X*, and [*X*] = concentration of free ligand. Therefore, the total concentration of ligand (free and bound), *X_t_* is given by
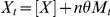
where *M_t_* is the bulk concentration of macromolecule in the active cell volume *V_cell_*. The total heat content *Q* of the solution in the active cell volume is
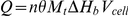
Taking into consideration, the volume change Δ*V_i_* accompanying the injection *i*, the heat released, Δ*Q_i_* from the *i* ^th^ injection is
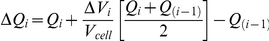
Binding free energy and entropy were obtained using the relation
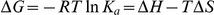
where R signifies the universal gas constant. Specific heat capacity changes ΔCp were subsequently derived from the plots of the binding enthalpy (ΔH), versus the experimental temperature (T), at constant pressure, using the following relation:
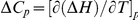



### Dynamic Light Scattering (DLS)

Dynamic light scattering measurements were performed on a Zetasizer Nano S particle analyzer from Malvern Instruments, UK. The light source was a He-Ne laser (632.8 nm) that utilizes 4 mW power at the same wavelength. Scattered light from the samples was collected at an angle of 173° and the intensity autocorrelation function was utilized to generate a correlation curve. Translational diffusion coefficients (D) were obtained from the homodyne autocorrelation function defined by:
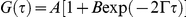
where *G*(*τ*) is the correlation coefficient, A is the amplitude of the correlation function, and B is the baseline.
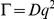
Where *D* is the Stokes-Einstein diffusion coefficient and *q* is the scattering vector.

Cumulants analysis of the correlation curve was used to obtain the intensity weighted mean hydrodynamic diameter or Z_av_ diameter of the ensemble of particles in the measurement window.

In order to study the effect of DST on the hydrodynamic size of soluble chromatin, dinucleosomes and trinucleosomes, the samples (300 µM mononucleotides) were treated with DST in drug to DNA base ratio of 0, 0.08, 0.16 and 0.25 at 25°C and the same were monitored by DLS. Diffusion coefficient values were calculated for each sample from the mean of the Z_av_ diameters obtained from 10 measurements.

### Electron Microscopy

Chromatin samples (soluble chromatin, di and trinucleosomes) were dialyzed against HEGN buffer (10 mM Hepes (pH 7.5), 0.25 mM EDTA, 10% glycerol, 15 mM NaCl). DST treatment was done at drug: DNA base ratios of 0, and 0.16 for 1 hour, at room temperature. Samples were fixed with 0.1% glutaraldehyde in HEGN buffer at 4°C for 16 hours, followed by extensive dialysis against HEGN buffer, for a total of 16 hours [31]. For spreading, samples were diluted to 20 µg/ml DNA using adsorption buffer (HEGN containing 2×10^−4^% BAC), and adjusted to room temperature for 30 minutes. 20 µl drops of sample were placed on freshly glow discharged 400 mesh carbon coated copper grids and allowed to adsorb for 5 minutes [32]. Excess sample was washed off by flotation on double distilled water. Grids were dehydrated in 98% ethanol for 3 seconds, air dried and rotary shadowed with platinum at an angle of 7° and pressure below 10^−4^ torr. Samples were examined using bright field optics in a TECNAI 12 SPIRIT BioTwin Transmission Electron Microscope (FEI, Netherlands) operating at 100 kV, and images were recorded on a CCD, Mega View III Soft Imaging System. The carbon coated copper grids contained 7 nm or 15 nm non-reactive nanospheres that served as internal standards.

### Statistical Analysis

60 images were acquired systematically for di and trinucleosomes, and 200–350 particles were selected for each sample. The internucleosomal (center-to-center) distance in dinucleosomes and trinucleosomes and the internucleosomal projection angle [30], [33] in trinucleosomes were then measured using Image J software [34]. A statistical analysis of the center-to-center distance and internucleosomal angle was performed using GraphPad Prism 5.0 software and the results were expressed in terms of the mean ± standard error of measurement. In order to estimate the statistical significance of the difference in mean ± SEM, the measured values of DST treated and untreated samples were compared by unpaired two-tailed t-test with Welch's correction [35]. The differences were considered significant when the p value was <0.05. Similar type of analysis has been reported earlier for chromatin structures visualized by scanning force microscopy [36], [37].

## Results

### Energetics of DST – chromatin interaction

**Figure 1:**
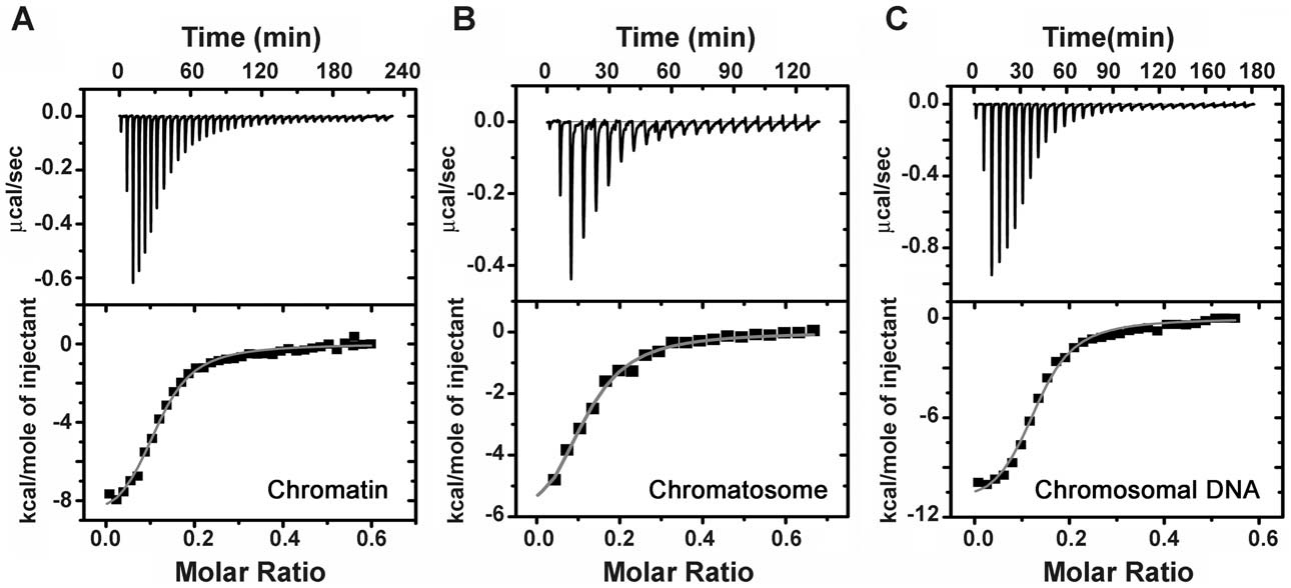
Representative isothermal titration calorimetry (ITC) profiles for the binding of DST to chromatin components. Titration profiles are shown for the interaction of DST with (A) soluble chromatin, (B) chromatosome and (C) chromosomal DNA. The experiments were performed in 5 mM Tris HCl (pH 7.4), 15 mM NaCl at 20°C.

**Figure 2:**
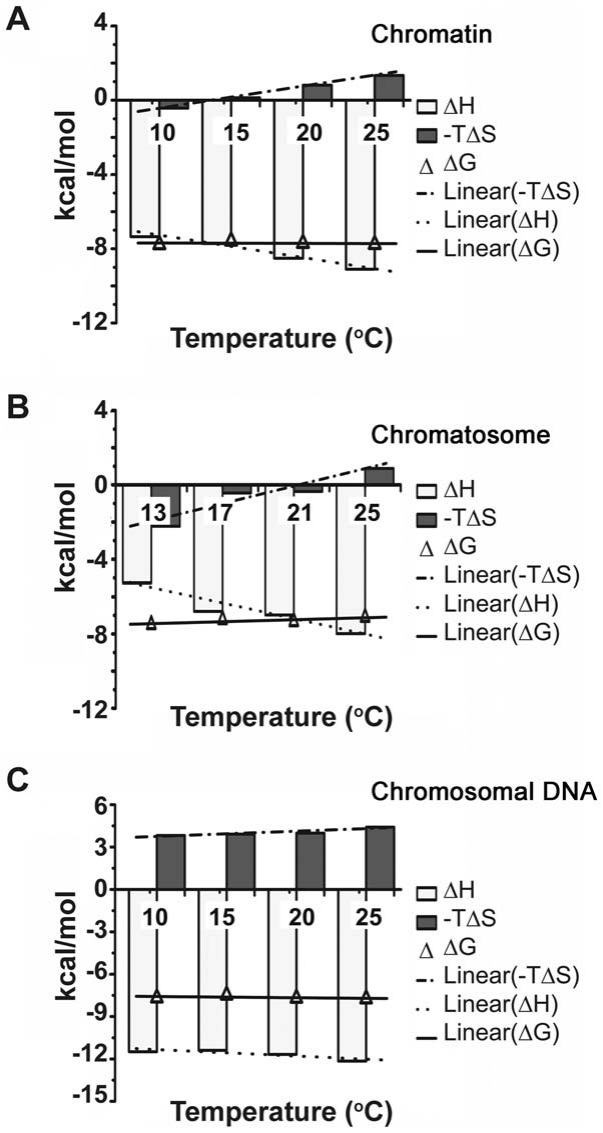
Energetics of the interaction of DST with chromatin components. The thermodynamic parameters (ΔH, −TΔS and ΔG) are plotted as function of temperature for the interaction of DST with (A) soluble chromatin, (B) chromatosome and (C) chromosomal DNA. All experiments were performed in 5 mM Tris HCl (pH 7.4), 15 mM NaCl.

Binding of DST to chromatin, and its components (chromatosome and chromosomal DNA) was quantitated by means of isothermal titration calorimetry (ITC). Representative thermograms for the titration are shown in Figure 1. Binding parameters obtained by ITC are summarized in Table 1. At 25°C, the apparent association constants (K_a_), for chromatin and chromosomal DNA are comparable. From titrations performed at four temperatures, the thermodynamic parameters (Figure 2) were evaluated. With increasing temperature, there is decrease in reaction enthalpy (ΔH) in all three cases, yielding negative values for the heat capacity change ΔCp (Table 1). Since, heat capacity values are indicative of structural change, an empirical relationship was obtained using the derivations of Spolar's group [38], [39]. It allowed estimation of the change in solvent accessible surface area (ΔSASA) of the macromolecules.

**Table 1:** Thermodynamic parameters for binding of DST with chromatin, chromatosome.

	Temperature (°C)	N (drugs/base)	ΔH (Kcal/mol)	ΔS (e.u)	K_a_(×10^5^)M^−1^	ΔG (Kcal/mol)	ΔCp (calK^−1^ mol^−1^)
Chromatin	10	0.15±0.002	−7.35±0.13	1.49	9.99±1.03	−7.77	−120.8±11.4
	15	0.15±0.002	−7.72±0.11	−0.465	5.63±0.38	−7.58	
	20	0.13±0.003	−8.51±0.31	−2.76	5.50±0.84	−7.70	
	25	0.14±0.002	−9.10±0.16	−4.48	4.89±0.37	−7.76	
Chromatosome	13	0.11±0.002	−5.26±0.15	7.79	5.25±0.57	−7.49	−208.7±42.9
	17	0.09±0.006	−6.78±0.60	1.51	2.74±0.52	−7.22	
	21	0.11±0.005	−6.98±0.38	1.21	2.80±0.32	−7.33	
	25	0.09±0.006	−7.98±0.62	−2.96	1.61±0.17	−7.10	
Chromosomal DNA	10	0.10±0.002	−11.49±0.38	−13.5	8.42±0.96	−7.67	−45.4±18.3
	15	0.12±0.004	−11.39±0.58	−13.6	4.53±0.83	−7.47	
	20	0.13±0.003	−11.68±0.29	−13.6	5.46±0.63	−7.69	
	25	0.14±0.002	−12.15±0.22	−14.8	4.75±0.38	−7.74	

a Experiments were performed in 5 mM Tris HCl (pH 7.4), 15 mM NaCl at the temperatures stated in the table.

The free energy change due to hydrophobic effect (ΔG_hyd_), is related to the change in specific heat capacity (ΔCp), by the following relation:
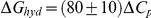


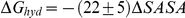
Therefore,
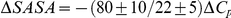
This indicates that large negative values of ΔCp are associated with the predominance of hydrophobic effect in any binding process. It is associated with the burial of solvent-exposed surface that leads to release of bound water. Similar approaches have been previously applied for Hoechst – DNA interaction [40], [41], and sanguinarine induced aggregation of chromatin [24]. For DST-chromatin interaction, it is implicit from our data that the association is accompanied by a finite amount of surface compaction, the extent of change being
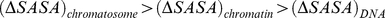
In case of systems possessing large negative ΔCp, the characteristic temperatures, T_H_ and T_S_ provide valuable information [42]. These define the temperature limits beyond which the reaction is governed solely by either the enthalpy or entropy factors. In between the limits, both the entropy and enthalpy factors come into play. Our calculations yield T_H_ and T_S_ for DST – chromatin interaction as −49.3°C and 13.8°C respectively. The same for DST –chromatosome are −13.3°C and 21.2°C respectively. Unlike chromatin and chromatosome, for chromosomal DNA, the reaction is mainly enthalpy driven and entropy-unfavorable at ambient temperatures. However, the free energy ΔG is found nearly constant in the temperature range studied.

### Hydrodynamic characterization of chromatin compaction

**Figure 3:**
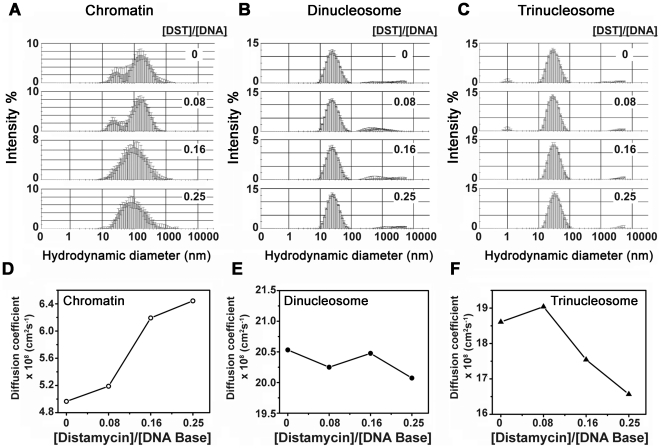
Dynamic Light Scattering (DLS) to study the influence of DST on the hydrodynamic properties of soluble chromatin, dinucleosomes and trinucleosomes. The intensity statistics of 10 measurements each are plotted for (A) soluble chromatin (300 µM DNA base), (B) dinucleosomes (300 µM DNA base) and (C) trinucleosomes (300 µM DNA base) in presence of increasing concentration of DST. Error bars indicate standard deviation. Diffusion coefficients calculated from Z_av_ radii are plotted as a function of DST concentration for (D) soluble chromatin, (E) dinucleosomes, and (F) trinucleosomes. All experiments were performed at 25°C.

We have employed dynamic light scattering (DLS) to study the influence of DST on the structure of chromatin in solution. DST causes compaction of soluble chromatin (Figure 3A), the Z_av_ diameter decreasing from 100.9 nm to 77.5 nm. Consequently, the diffusion coefficient (Figure 3D) increases from 4.9×10^8^ cm^2^ s^−1^ to 6.4×10^8^ cm^2^ s^−1^. Chromatin compaction is believed to occur as a result of changes in the geometry of its linker DNA and internucleosomal angle [43]. We have, therefore, performed similar experiments with dinucleosomes and trinucleosomes, with a view to understand the roles of linker DNA and the internucleosomal angle in the compaction process. Dinucleosomes possess a single linker DNA and trinucleosomes, a single internucleosomal angle [30], [44]–[47]. DLS measurements of dinucleosomes (Figure 3B) demonstrate very little change. The Z_av_ diameter fluctuates between 24.3 nm and 24.8 nm. Diffusion coefficients, derived from Z_av_ values, reflect a similar trend (Figure 3E). Hence the linker DNA appears to be unperturbed by DST. It agrees with previous findings of Marion *et al.*, and Bednar *et al.*, who studied salt induced compaction of chromatin [48], [49]. For trinucleosomes (Figure 3C), initially there is a minor decrease in Z_av_ diameter from 26.8 nm to 26.2 nm. Increase in the DST input ratio, results in an increase in Z_av_ diameter upto 30.1 nm. Consequently, the diffusion coefficient (Figure 3F) changes from 18.6×10^8^ cm^2^ s^−1^ at DST to DNA ratio of 0 to 19×10^8^ cm^2^ s^−1^ at DST to DNA ratio of 0.08 and 16.5×10^8^ cm^2^ s^−1^ at DST to DNA ratio of 0.25. However, the peak position remains almost invariant with DST concentration.

It seems unusual, that a change in chromatin structure is not reflected in dinucleosomes and trinucleosomes, which supposedly represent the internucleosomal distance and angle parameters respectively. However, keeping in mind the fact, that DLS measurements yield the apparent size of a solvated, dynamic particle, it is possible that the change in geometry of dinucleosomes and trinucleosomes is masked by their outer hydration shell, and hence, not detected by DLS. Chromatin, on the other hand, is a multimer of nucleosomes. So, in case of chromatin, the extent of structural change is relatively higher, due to cumulative changes in many internucleosomal distances and angles. As a result, the masking effect of hydration is alleviated, and a change in Z_av_ diameter is detectable.

### Basis of structural alterations in higher order chromatin

**Figure 4:**
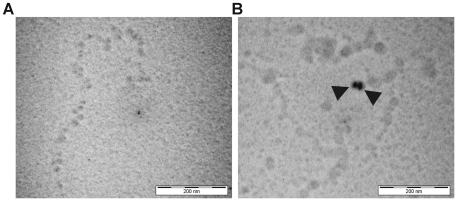
Electron microscopy of soluble chromatin. Chromatin samples were incubated with DST in drug to DNA base ratio of 0.16 and processed as detailed under “Materials and Methods”. (A) Soluble chromatin incubated with buffer for 1 hour. (B) Soluble chromatin incubated with DST under similar experimental conditions. Black arrowheads indicate 15 nm nanosphere standards and the scale bar indicates 200 nm.

**Figure 5:**
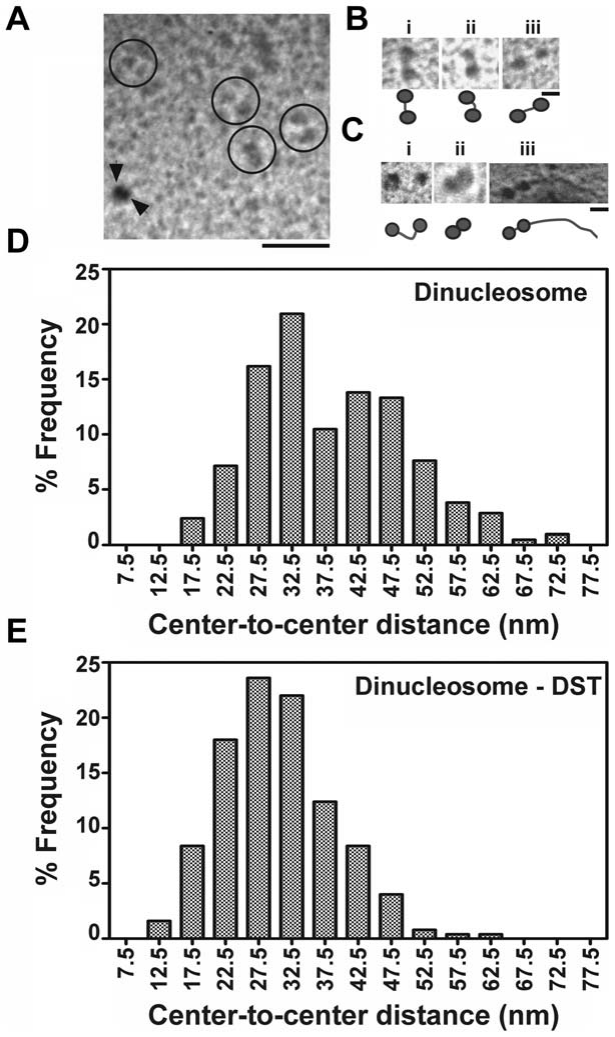
Analysis of dinucleosome morphology. (A) Survey view of a glutaraldehyde fixed dinucleosome fraction, shadowed with platinum. Some clearly defined dinucleosomes have been encircled. Black arrowheads indicate 15 nm nanosphere standards and scale bar indicates 100 nm. Three representative dinucleosomes are shown in higher magnification in (B). Scale bar indicates 20 nm. Three representative DST treated dinucleosomes are shown in (C). Scale bar is 20 nm. (D, E) Statistical analysis of the center to center distances of dinucleosomes. The percentage frequency of particles is plotted against the center to center distance in (D) free dinucleosomes and (E) DST treated dinucleosomes. Frequency distributions were obtained for 5 nm bin size. The ratio of DST to DNA base was 0.16.

**Figure 6:**
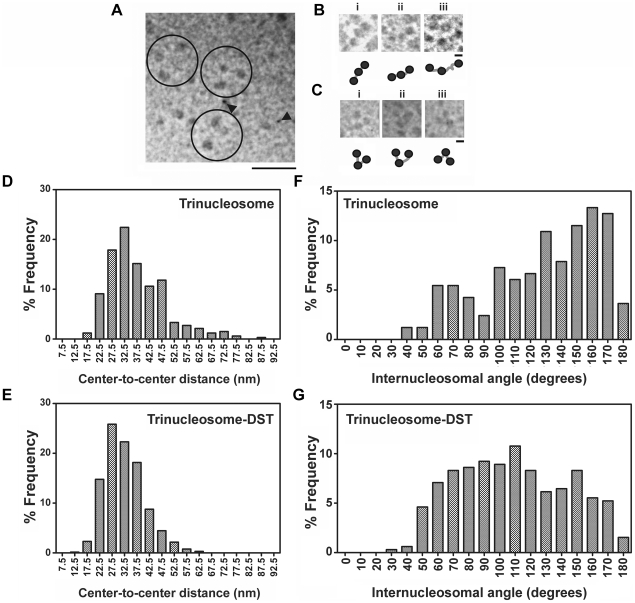
Analysis of trinucleosomes morphology. (A) Survey view of a glutaraldehyde fixed trinucleosome fraction, shadowed with platinum. Some clearly defined trinucleosomes have been encircled. Black arrowheads indicate 7 nm nanosphere standards and scale bar indicates 100 nm. Three representative trinucleosomes are shown in higher magnification in (B). Scale bar indicates 20 nm. Three representative DST treated trinucleosomes are shown in (C). Scale bar is 20 nm. (D, E) Statistical analysis of the center to center distances of trinucleosomes. The percentage frequency of particles is plotted against the center to center distance in (D) free trinucleosomes and (E) DST treated trinucleosomes. Frequency distributions were obtained for 5 nm bin size. (F,G) Statistical analysis of the internucleosomal angles of trinucleosomes. The percentage frequency of particles was plotted against the internucleosomal projection angle in (F) free trinucleosomes and (G) DST treated trinucleosomes. The bin size for the frequency distribution was 10 degrees. DST treatment was performed at drug to DNA base ratio of 0.16.

Both ITC and DLS showed compaction of soluble chromatin. We have therefore used electron microscopy to elucidate the alteration in geometry of DST treated chromatin as compared to untreated one. Electron micrographs of free and DST treated soluble chromatin are shown in Figure 4. Indications of compaction are apparent at drug to DNA base ratio of 0.16. Electron micrographs of free and DST treated dinucleosomes are shown in Figure 5 (B, C). DST treatment of dinucleosomes leads to compaction. An important observation in this regard is the appearance of dinucleosomes with bent linker DNA (Figure 5C i). Frequency histogram of center-to center distances of dinucleosomes (Figure 5D) reveals the existence of heterogeneity in linker lengths with two apparent maxima at ∼32.5 nm and ∼42.5 nm. Upon DST treatment, the heterogeneity of linker lengths is considerably reduced and the frequency histogram (Figure 5E) shows a single peak at ∼27.5 nm. Quantitative analysis of the dinucleosome center-to-center distances reveal that the mean value decreases from 38.5±0.8 nm in free dinucleosomes to 30.5±0.5 nm in DST treated ones. According to the t-test performed, this difference is statistically significant at 95% confidence level (with obtained p value<0.0001). Electron micrographs of free and DST treated trinucleosomes are shown in Figure 6(B,C). The frequency histogram of center-to center distances for free trinucleosomes (Figure 6D) apparently peaks at ∼32.5 nm with a second population at ∼47.5 nm. For DST treated trinucleosomes (Figure 6E), the peak population shifts to 27.5 nm. The mean value however decreases from 37.4±0.6 nm to 32.6±0.3 nm, which is statistically significant at 95% confidence level (p value obtained <0.0001). In case of the internucleosomal projection angle of trinucleosomes, the frequency distribution (Figure 6 F, G) clearly reveals contraction of internucleosomal angle upon DST treatment. This change is reflected in the mean values, which decreases from 127.7±2.9 degrees to 108.4±1.9 degrees. Likewise, the t-test for comparison of measured angles also indicates statistical significance at 95% confidence level (p value obtained <0.0001).

## Discussion

Interaction of the minor groove binder DST, with short double stranded DNA has been widely studied [1], [2], [7], [8], [9], [ and 11]. But the effect of the same on DNA, wound in chromatin, has not been truly visited. We present herein results that elucidate how DST induced perturbations in the minor groove cause the chromatin architecture to change.

In order to characterize the binding of DST with chromatin from a thermodynamic perspective, we have chosen three systems – soluble chromatin, chromatosome and chromosomal DNA. Soluble chromatin, isolated from rat liver, resembles physiological chromatin. Chromatosome and chromosomal DNA templates are expected to account for the binding of DST to histone wrapped DNA, and protein free DNA respectively. It is to be noted that the DNA component in all three systems are similar. This ensures that the difference in thermodynamic parameters obtained, does not arise from difference in DNA sequences [13]. A comparative study of the three systems would therefore help to identify the preferred ligand binding site in chromatin. Results from ITC reveal comparable values of the binding constant K_a_ for chromatin and chromosomal DNA. It can be inferred that the binding site for DST is equally accessible in case of chromatin and chromosomal DNA. This is consistent with a recent finding, that the minor groove of nucleosomal DNA accommodates pyrrole-immidazole polyamides, while retaining the integrity of histone-DNA interactions [50]. This is further supported by nearly similar site sizes for chromatin and chromosomal DNA. Interestingly, the free energy (ΔG) of binding of DST to chromatin, and chromosomal DNA are also similar. However, DST-chromosomal DNA interaction is mainly enthalpy driven, with an unfavorable entropy contribution. DST-chromatin system on the contrary presents enthalpy-entropy compensation, a common feature of biological interactions [13]. The slightly lower binding free energy (ΔG) for DST- chromatosome system may be attributed to the lack of enthalpically favorable binding at the linker DNA. Temperature dependent studies have been utilized to obtain the change in heat capacity (ΔCp), which is correlated with the change in solvent accessible surface area (SASA), and hence the nature of conformational alteration. These studies reveal the existence of a positive ΔSASA in all three cases, and have singled out chromatosomes as the system, where the heat capacity change ΔCp, for DST binding is the largest. ΔCp value corresponding to DST – chromatin association also indicates contraction of surface area, leading to compaction.

It may be noted here, that earlier studies of DST involved nucleosome core particles, reconstituted on either *tyr*T DNA fragment or on cloned sequences of synthetic DNA with phased (A/T)_4_ stretches [21], [22]. In such experiments, reconstitution was essential in order to maintain sequence homogeneity of template DNA. However, the thermodynamic similarity of such reconstituted nucleosomal particles with soluble chromatin has not yet been confirmed. On the contrary, the use of soluble chromatin for characterization of binding of small molecules, has been well established [24], [27], [28], [51]–[54]. Furthermore, the low fidelity of reconstitution reactions, limits the yield of sample, and their use in biophysical experiments that require large sample amounts. Hence our experiments were based on chromatin from natural source.

Indications of compaction obtained from the change in solvent accessible surface area, led us to characterize the ligand induced structural changes at the chromatin level. Dynamic light scattering was used to investigate the structural changes in a hydrated context. DLS indicates that the hydrodynamic diameter of bulk soluble chromatin decreases upon DST treatment in a concentration dependent manner. The concentration dependence implies that it occurs as a result of the association of chromatin with DST. This finding prompted us to probe the determinants of the chromatin folding phenomenon. Such compaction is hypothesized to occur by either of two mechanisms – linker DNA bending and internucleosomal angle contraction. The mechanism adopted, depends on the structure of the chromatin compact state [43], [55], [56]. We have, therefore, investigated the effect of DST on the conformation of linker DNA and the internucleosomal angle. The simplest systems to study linker DNA and internucleosomal angle are dinucleosomes and trinucleosomes respectively. However, in both dinucleosomes and trinucleosomes, our DLS results indicate minor change in the Z_av_ diameter and consequently, the diffusion coefficient.

It may be noted here, that intensity based DLS measurements are biased towards sample population of larger hydrodynamic size, even if they are present in statistically insignificant amounts. This is because Rayleigh scattering is proportional to the sixth power of hydrodynamic radius. Hence, minor changes in the hydrodynamic diameter of dinucleosomes and trinucleosomes may not be detected by DLS. Transmission electron microscopy, on the contrary, highlights the statistically significant consequences of DST association, and hence would render more reliable results.

Electron micrographs of soluble chromatin show DST induced compaction. This agrees with the results obtained from DLS. From the electron micrographs of dinucleosomes and trinucleosomes, it is apparent that mechanistically, the compaction occurs via both bending of linker DNA and contraction of internucleosomal angle. Frequency histograms of center to center distances obtained from EM, also suggest a reduction in the population heterogeneity upon DST treatment.

This is consistent with the presently reported change in specific heat capacity, ΔCp, and ΔSASA, accompanying DST association. Reduction in solvent exposed surface area is probably achieved by pulling in of the linker arms towards the nucleosome core. It leads to the reduction of internucleosomal distance and the internucleosomal angle as well. Moreover, the reduced population heterogeneity of di and trinucleosomes is a direct consequence of free energy minimization upon interaction with DST.

Compaction of chromatin is a phenomenon that modulates the recognition of genes towards transcription factors [57]. For a groove binder like DST, it is likely, that it's binding to chromatin, influences the torsional state of the DNA therein. Consequently, the twist registry of consecutive nucleosomes favours compaction [58]. Since DST binding to the minor groove would adversely affect protein binding to the major groove [16], the observed effects of DST upon gene regulation [14]–[20], may be intimately related to its effect on chromatin structure.

In conclusion, we report here the thermodynamic characteristics of the association of DST with various structural levels of chromatin. Indications of structural change obtained thereof have been validated by two complementary methods – DLS and TEM. Both methods show compaction of chromatin in presence of DST. A statistical analysis of TEM results with dinucleosome and trinucleosome indicate that the compaction occurs by both linker bending and a reduction in the internucleosomal angle.

Overall, this work derives its relevance from the fact that it is the first in-depth report of the binding of DST with chromatin. It addresses an important issue of how the structure of chromatin changes in presence of DNA binding ligands, and attempts to comprehend structural changes from an energetic perspective.
